# Supplementary material for: State-of-the-Art Native Mass Spectrometry and Ion Mobility Methods to Monitor Homogeneous Site-Specific Antibody-Drug Conjugates Synthesis
Source: Pharmaceuticals (Basel). 2021 May 24;14(6):498. doi: 10.3390/ph14060498 (PMC8225019; doi:10.3390/ph14060498)
Supplement: Supplementary file 1 [file pharmaceuticals-14-00498-s001.zip › pharmaceuticals-1174280-supplementary.pdf]

pharmaceuticals

SUPPLEMENTARY INFORMATION

State of the Art Native Mass Spectrometry and Ion Mobility Methods to Monitor Homogeneous Site-Specific Antibody-Drug Conjugates Synthesis.

Evolène Deslignière[1,2], Anthony Ehkirch[1,2], Bastiaan L. Duivelshof[3,4], Hanna Toftevall[5], Jonathan Sjögren[5], Davy Guillarme[3,4], Valentina D'Atri[3,4], Alain Beck[6], Oscar Hernandez-Alba[1,2] and Sarah Cianférani[1,2]*

[1] Laboratoire de Spectrométrie de Masse BioOrganique, IPHC UMR 7178, Université de Strasbourg, CNRS, 67087 Strasbourg, France

[2] Infrastructure Nationale de Protéomique ProFI – FR2048, 67087 Strasbourg, France

[3] School of Pharmaceutical Sciences, University of Geneva, CMU – Rue Michel-Servet 1, 1211 Geneva 4, Switzerland

[4] Institute of Pharmaceutical Sciences of Western Switzerland, University of Geneva, CMU – Rue Michel-Servet 1, 1211 Geneva 4, Switzerland

[5] Genovis AB, SE-220 07 Lund, Sweden

[6] IRPF - Centre d'Immunologie Pierre-Fabre (CIPF), 74160 Saint-Julien-en-Genevois, France

* Correspondence: sarah.cianferani@unistra.fr

TABLE OF CONTENTS:

- **Figure S1:** Online SEC-nMS analysis of T-DM1.
- **Figure S2:** Online SEC-nMS analysis of T0, T1 and T2 after thermal stress.
- **Table S1:** $^{TW}CCS_{N2}$ measurements of intact and IdeS-digested reaction products.
- **Figure S3:** CIU experiments at the intact level for the 23+ charge state.
- **Table S2:** RMSDs between technical triplicates for CIU fingerprints at the intact level for 23+ and 24+ charge states.

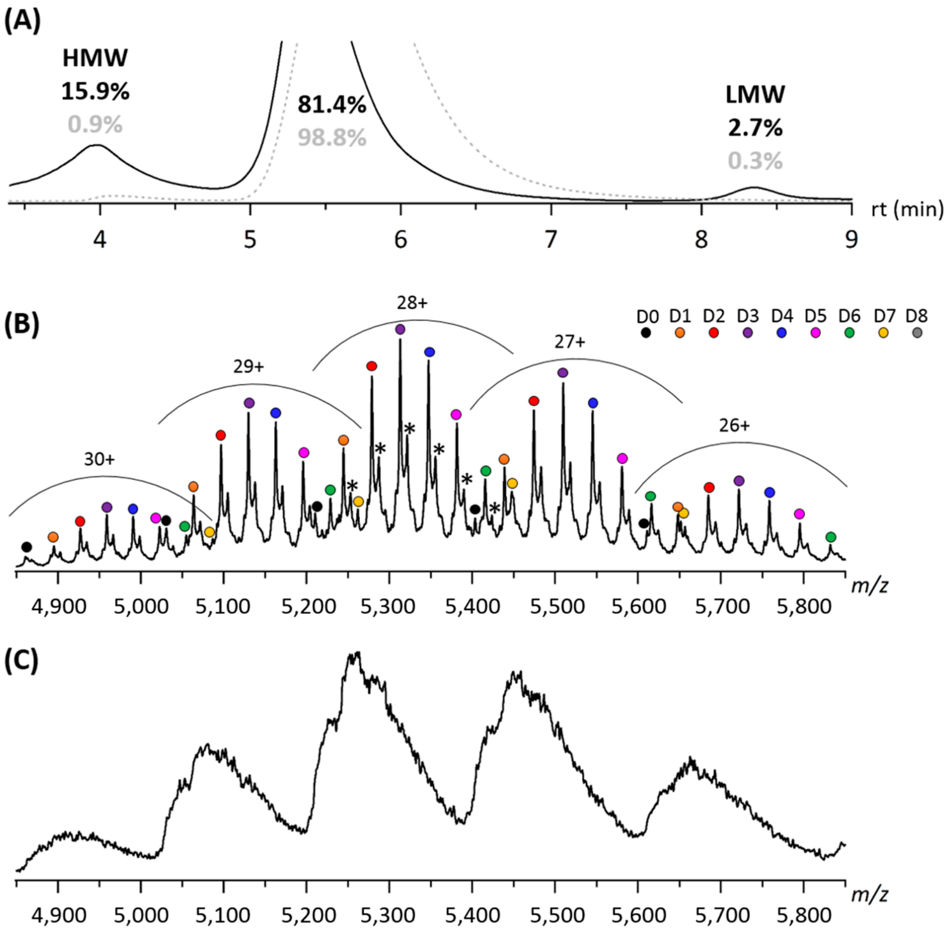

Figure S1. Online SEC-nMS analysis of T-DM1. (A) Overlaid SEC chromatograms of stressed (solid line) and non-stressed (dotted line, grey) samples. (B) SEC-nMS spectrum of intact non-stressed T-DM1 with corresponding UV chromatogram (280 nm) depicted in inset; ✱ = linker adducts (+220 Da). (C) SEC-nMS spectrum of thermally-stressed T-DM1.

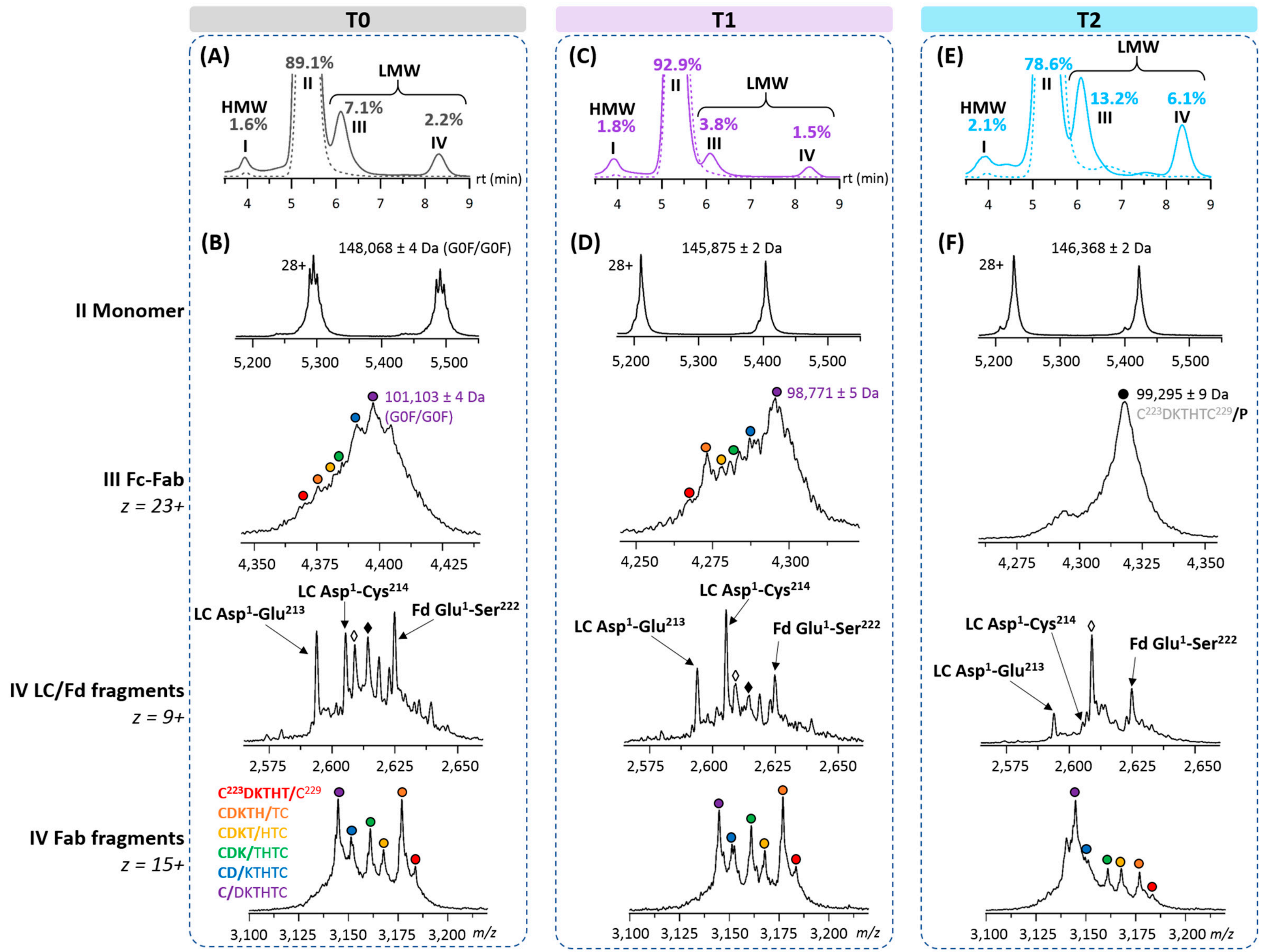

Figure S2. Online SEC-nMS analysis of T0, T1 and T2 after thermal stress. (A, C, E) Overlaid SEC chromatograms of stressed (solid line) and non-stressed (dotted line) samples. Relative amounts of HMWS and LMWS are given for the stressed sample; I = HMW dimers, II = main product, III = Fc-Fab fragments and IV = LC, Fd and Fab fragments. (B, D, F) SEC-nMS spectra of monomers and hinge-related fragments generated upon thermal stress. ◊ = sulfurized Cys^{214} (+32 Da compared to LC Asp^{1}-Cys^{214}). ♦ = +SO_2H on Cys^{214} (+80 Da compared to LC Asp^{1}-Cys^{214}).

Table S1. $^{TW}CCS_{N2}$ measurements of intact and IdeS-digested reaction products. ¶ Mass-based estimation of CCS, CCS = 2.435 × $MW^{2/3}$ according to Ruotolo *et al.* (*Nat Protoc* **2008**, 3(7), 1139-1152).

		$^{TW}CCS_{N2}$ (nm²)			
		**T0**	**T1**	**T2**	**T-GlyCLICK-DM1**
**Intact**	**Predicted¶**	68.1	67.5	67.6	68.4
	**23+**	73.8 ± 0.2	73.4 ± 0.2	73.6 ± 0.2	74.3 ± 0.2
	**24+**	75.3 ± 0.2	74.9 ± 0.1	75.1 ± 0.1	75.9 ± 0.1
**Fc fragment**	**Predicted¶**	33.3	32.3	32.5	33.6
	**12+**	34.1 ± 0.1	33.2 ± 0.1	33.4 ± 0.1	34.6 ± 0.2
	**13+**	35.2 ± 0.1	34.4 ± 0.1	34.7 ± 0.1	35.9 ± 0.1
**F(ab')$_2$ fragment**	**Predicted¶**	51.6	51.6	51.6	51.6
	**20+**	56.8 ± 0.1	56.8 ± 0.1	56.6 ± 0.2	57.0 ± 0.1
	**21+**	58.2 ± 0.1	58.2 ± 0.1	58.0 ± 0.2	58.2 ± 0.1

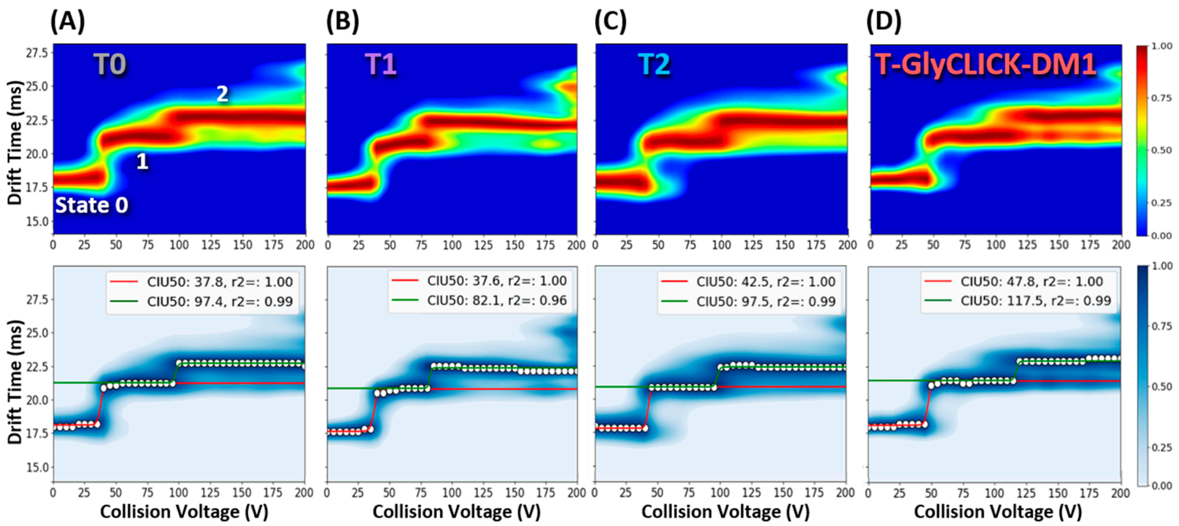

Figure S3. CIU experiments at the intact level for the 23+ charge state. CIU fingerprints (upper panel) and CIU50 analysis (lower panel) were acquired to compare the resistance to gas-phase unfolding of the reaction compounds (A) T0, (B) T1, (C) T2 and (D) T-GlyCLICK-DM1.

Table S2. RMSDs between technical triplicates for CIU fingerprints at the intact level for 23+ and 24+ charge states.

	RMSD between technical replicates (n = 3)			
	**T0**	**T1**	**T2**	**T-GlyCLICK-DM1**
23+	13.3	7.3	11.2	8.4
24+	9.7	5.1	11.2	7.9
